# Cyclooxygenase-2 inhibition: effects on tumour growth, cell cycling and lymphangiogenesis in a xenograft model of breast cancer

**DOI:** 10.1038/sj.bjc.6603593

**Published:** 2007-02-06

**Authors:** N L P Barnes, F Warnberg, G Farnie, D White, W Jiang, E Anderson, N J Bundred

**Affiliations:** 1 Department of Academic Surgery, South Manchester University Hospital, Research and Education Building 2nd Floor, Southmoor Road, Wythenshawe, Manchester M23 9LT, UK; 2 The Department of Angiogenesis and Metastasis Research, University of Wales College of Medicine, Cardiff, Wales, UK

**Keywords:** breast, cyclooxygenase-2, apoptosis, lymphangiogenesis

## Abstract

Cyclooxygenase-2 (COX-2) is associated with poor-prognosis breast cancer. We used a nude mouse xenograft model to determine the effects of COX-2 inhibition in breast cancer. Oestrogen receptor (ER)-positive MCF7/HER2-18 and ER-negative MDAMB231 breast cancer cell lines were injected into nude mice and allowed to form tumours. Mice then received either chow containing Celecoxib (a COX-2 inhibitor) or control and tumour growth measured. Tumour proliferation, apoptosis, COX-2, lymphangiogenesis and angiogenesis were assessed by immunohistochemistry (IHC), Western blotting or Q-PCR. Celecoxib inhibited median tumour growth in MCF7/HER2-18 (58.7%, *P*=0.029) and MDAMB231 (46.3%, *P*=0.0002) cell lines compared to control. Cyclooxygenase-2 expression decreased following Celecoxib treatment (MCF7/HER2-18 median control 65.3% *vs* treated 22.5%, *P*=0.0001). Celecoxib increased apoptosis in MCF7/HER2-18 tumours (TUNEL 0.52% control *vs* 0.73% treated, *P*=0.0004) via inactivation of AKT (median pAKT^ser473^ 57.3% control *vs* 35.5% treated, *P*=0.0001 – confirmed at Western blotting). Q-PCR demonstrated decreased podoplanin RNA (lymphangiogenesis marker) in the MCF7/HER2-18 – median 2.9 copies treated *vs* 66.6 control (*P*=0.05) and MDAMB231-treated groups – median 160.7 copies *vs* 0.05 control copies (*P*=0.015), confirmed at IHC. Cyclooxygenase-2 is associated with high levels of activated AKT^ser473^ and lymphangiogenesis in breast cancer. Cyclooxygenase-2 inhibition decreases tumour growth, and may potentially decrease recurrence, by inactivating AKT and decreasing lymphangiogenesis.

Cyclooxygenase-2 (COX-2) is overexpressed in approximately 70% of cases of *in situ* and 60% of invasive breast cancer (Boland *et al*, 2004). The COX genes encode for enzymes that catalyse the rate-limiting step of conversion from arachidonic acid to prostaglandins. Two main isoforms, COX-1 and COX-2, were first demonstrated in the early 1990s (Fu *et al*, 1990; Xie *et al*, 1991). Cyclooxygenase-1 is constitutively expressed in most tissues, and is responsible for physiological housekeeping functions such as gastric cytoprotection, regulation of renal blood flow and platelet aggregation (Smith *et al*, 2000). Cyclooxygenase-2 is an inducible isoform, and is expressed by cells involved in the inflammatory process (Subbaramaiah *et al*, 1996; Smith *et al*, 2000) (i.e. macrophages, monocytes, synoviocytes) and also in a wide variety of solid epithelial tumours (Gasparini *et al*, 2003). The increased expression of COX-2 in tumourigenesis is secondary to multiple activations and as a response to growth factors and oncogenes, including those in the EGFR/HER2/RAS/MAP kinase pathway. This latter pathway is of particular interest in breast cancer as HER2 is often upregulated in both invasive and *in situ* disease and is associated with poor prognosis tumours (Slamon *et al*, 1987; Sposto and Silverstein, 2002). Conversely, COX-2 is rarely expressed in normal tissue (unless in proximity to an area of neoplasia or inflammation). When COX-2 is expressed in normal tissue, it has been found in a subpopulation of normal breast cells with silenced p16^ink4a^ (which have been postulated as precursors of breast cancer) (Crawford *et al*, 2004).

Studies of transgenic mice engineered to overexpress COX-2 have shown that these mice form mammary tumours earlier and more often than wild-type mice, their mammary glands contain fewer apoptotic cells and show reduced expression of the proapoptotic proteins BAX and BCl-x(L) (Liu *et al*, 2001). Elevated COX-2 expression leads to increased tumour recurrence and decreased survival in invasive breast cancer (Crawford *et al*, 2004). The recent publication of the Women's Health Initiative results (Harris *et al*, 2003) suggest that the regular use of nonsteroidal anti-inflammatory drugs (NSAIDs) has a significant chemo-preventative effect against developing breast cancer, with a 28% reduction (relative risk 0.72, 95% CI 0.56–0.91) with regular NSAID use for 10 years or more.

Previous studies have shown significant decreases in various solid tumour growths following the administration of COX-2 inhibitors (Connolly *et al*, 2002; Howe *et al*, 2002), although there is still a need to clarify the mechanism of action in breast cancer and identify surrogate markers of effect.

The aims of this study were to use a xenograft model of breast cancer to confirm the effect of COX-2 inhibition on tumour growth *in vivo*, and to subsequently determine the mechanism of action, by studying expression of markers of cell proliferation (Ki67 and p21^cip1^), apoptosis (TUNEL) and AKT activation.

We also aimed to determine whether Celecoxib perturbed the production of the new lymphatic vessels (lymphangiogenesis). It has recently been demonstrated that expression of the lymphangiogenic factor VEGF-C is significantly elevated in cells overexpressing COX-2 (Su *et al*, 2004). The production of new lymphatic vessels by the tumour is a key step in the promotion of cancer metastasis and COX-2 inhibitors have been shown to have antimetastatic activity in a metastatic mouse model (Roche-Nagle *et al*, 2004). In addition, markers of tumour endothelial cell formation have been shown to correlate with prognosis in breast cancer (Davies *et al*, 2004). In this paper, we used podoplanin, a marker of lymphatic endothelial cells to demonstrate changes in lymphangiogenesis following treatment with Celecoxib in our xenograft model.

## MATERIALS AND METHODS

### Tumour cell lines

One oestrogen receptor (ER)-positive and one ER-negative breast cancer cell line were chosen for study. MCF7/HER2-18, is an ER-positive MCF7 cell line which had been stably transfected with HER2 (EGFR negative /HER2 positive/ER positive), a gift from Professor CC Benz, University of California San Francisco, and MDAMB231 is an ER-negative breast cancer cell line which is EGFR positive /HER2 negative. All cells were routinely maintained in Dulbecco's Modified Eagle Medium with 10% foetal calf serum, 2 mM glutamine, 100 U penicillin and 100 *μ*g streptomycin per 500 ml. The cells were incubated at 37°C in a humidified environment and passaged at near confluence.

### Nude mouse xenograft model

The experimental design followed a well-established female nude mouse model from our department (Gandhi *et al*, 2000; Chan *et al*, 2002). Following ethical approval, 2–4 million cells were injected into each flank of female BLAB/c nu/nu mice, aged 6–8 weeks, and allowed to form tumours. The tumours were measured with callipers twice weekly (length × width) and the mice weighed. The mice had free access to feed, water and bedding at all time, and were housed with a 12 h light/dark cycle in filter top cages, maximum six mice per cage.

Tumour volumes (mm^3^) were calculated by the formula length × length × (width/2). When >75% of the tumours were palpable the mice were randomised to receive either chow containing 0.15% Celecoxib (Celecoxib donated from Pfizer/chow constituted at Purina Mills, USA) or control chow. The tumours continued to be measured and the mice weighed twice weekly. When tumours in either group reached 1 cm^3^, the experiment was ended in accordance with UK Home Office guidelines and the tumours were harvested. The tumours were then split, with half fixed in 4% formalin and embedded in paraffin blocks for immunohistochemistry (IHC), and the remainder snap frozen in liquid nitrogen.

### Immunohistochemistry

Three micron sections of the paraffin blocks were cut onto APES-coated slides. The slides were dewaxed in xylene and rehydrated through graded alcohols to PBS. Staining for COX-2 and Ki67 (Boland *et al*, 2004) was as described previously. All incubations were carried out at room temperature unless otherwise stated. For p21, pAkt^ser473^ and podoplanin, endogenous peroxidase activity was blocked by immersing slides in 0.2% H_2_O_2_ in methanol for 10 min. At TUNEL staining, sections were treated with proteinase K for 15 min before quenching endogenous peroxidase activity. Antigen retrieval was performed by immersing slides in citrate buffer (pH 6), in a pressure cooker for p21 (120 s), pAkt^ser473^ (100 s) and podoplanin (120 s).

### TUNEL

Sections were incubated in equilibration buffer (Apoptag Kit, Intergen, Norcross, GA, USA) for 1 h. Then working strength TdT enzyme was applied to the sections, which were incubated for 1 h at 37°C. Immersing the sections in working strength stop buffer for 10 min halted the reaction. Following washing in PBS, the sections were incubated with anti-digoxigenin conjugate for 30 min.

### p21^cip1^

Sections were incubated with the Mouse-on-Mouse (MOM) kit blocking solution with avidin for 1 h. Following washing in PBS, the sections were incubated for 5 min with the working solution of MOM dilutent for 5 min, and then incubated with the primary mouse anti-p21 antibody (DAKO M7202 mouse monoclonal) 1 : 25 made up in MOM dilutent with biotin for 60 min. Following washing in PBS, sections were incubated with working MOM kit biotinylated anti-mouse IgG for 30 min.

### Phosphorylated Akt^ser473^

Nonspecific binding was blocked with 10% normal goat serum in PBS with avidin for 15 min. The sections were then incubated with the primary phosphor-AKT/PKB^ser473^ antibody (rabbit polyclonal, Cell Signalling Technology, Danvers, MA, USA, #9277) 1 : 30 in 10% normal goat serum with biotin for 2 h, then with the biotinylated goat anti-rabbit secondary 1 : 200 (Vector BA1000) in 10% normal goat serum for 1 h.

### Podoplanin

Nonspecific binding was blocked by incubating slides in 10% normal rabbit serum in PBS with avidin for 30 min, the slides were then incubated with the primary monoclonal antibody to podoplanin (ab11936, AbCam) 1 : 500 made up in 10% normal rabbit in PBS with biotin for 2 h. After washing in PBS, the biotinylated rabbit anti-hamster secondary was added (1 : 500 ab6782 AbCam) for 1 h.

For p21, pAkt^ser473^, and podoplanin, following the secondary antibody, sections were incubated with Vectastain Elite ABC reagent (Vector, Peterborough, UK) for 30 min. All stainings were visualised with DAB, counter-stained with haematoxylin, slides then dehydrated, cleared and mounted.

### IHC scoring

All scorings were carried out blind to the treatment group. For Ki67, p21^cip1^ and pAkt^ser473^, the percentage of positive nuclei out of at least 1000 counted, was determined over representative tumour areas. At least 3000 cells were counted when determining the TUNEL score, as there were few apoptotic cells in each specimen. Reproducibility and accuracy of the scoring was ensured by repeat scoring and multiple observer scoring of the initial samples. For pAkt^ser473^ the cytoplasmic staining intensities were scored on a rating 0–3+. The COX-2 cytoplasmic staining was scored on both the percentage of positive cells and the staining intensity for the MCF7/HER2-18. The grading of intensity for each section was determined following reference to sections taken to be representative for each grade of intensity; 0=no staining, 1=mild staining, 2=moderate staining, 3=strong, 4=very strong. At least 1000 cells were also counted for each section, and the percentage of positively staining cells determined, and assigned a numerical score; 1=no staining, 2=<10%, 3=11–50%, 4⩾50%. It was considered inappropriate to use the intensity score for the MDAMB231 cells, as COX-2 expression was localised to cytoplasmic vesicles, and there was no discernable difference in intensity. Scoring was therefore calculated from the proportion of cells containing cytoplasmic vesicles. All scorings were carried out using a light microscope, grid graticule and cell counter at × 400 magnification. For podoplanin, vessel density was calculated using a Chalkley point graticule. Scoring was taken as the median number of vessels crossing the points, from six separate fields of high vessel density, at × 400 magnification.

### Western blotting

Snap frozen tumour samples were utilised for Western blotting. Crushing the tissue samples in liquid nitrogen created tumour powder. Lysis buffer was then added to create cell lysates, which were left on ice for 40 min before centrifugation (at 13 000 r.p.m. for 10 min at 4°C) and protein estimation (using the Biorad protein assay solution and a GeneQuant pro machine).

Following the addition of sample buffer to the lysates, 50 *μ*g of protein was resolved onto SDS gel and transferred to Hybond membranes using a TRIS/glycine/SDS electrode tank buffer, run overnight. Membranes were blocked with 5% skimmed milk–TBS-Tween for 1 h and then probed with the primary antibodies; pPKB/pAKT^ser473^ (1 : 1000 #9271 Cell Signaling Technology) and then total PKB/AKT (1 : 1000 #9272 Cell Signaling Technology) as a loading control.

After washing in TBS-Tween, the membranes were incubated with a donkey anti-rabbit HRP-linked secondary antibody (1 : 1000 Amersham Biosciences NA934V, Piscataway, NJ, USA) for 1 h. Following thorough washing in TBS-Tween for 6 × 10 min, the level of specific protein was visualised by chemiluminescence (Supersignal West Dura extended duration substrate; Pierce Biotechnology, Rockford, IL, USA). Both total PKB/AKT and pPKB/AKT^ser473^ bring up single bands of 60 kDa. Quantification of protein levels was estimated at subsequent densitometry using GeneTools from Syngene version 3.05.

## Q-RT-PCR

The assessment of lymphangiogenesis (podoplanin) and angiogenesis (CD31) was determined by quantitative PCR utilising snap-frozen tumour samples, which were stored in liquid nitrogen until use. Samples were homogenised to extract RNA using RNA-Zol reagent (Abgene homogenisation kit, Epsom, UK) as per manufacturer's instructions. cDNA was subsequently generated from 1 *μ*g of RNA using the Enhanced Avian Reverse Transcriptase kit (Abgene, UK). Levels of podoplanin and CD31 transcripts were estimated using the Ampliflour Uni Primer detection kit (Intergen, UK) and 2 × Q-PCR master mix according to manufacturers instructions. Quantification was carried out using the iCycler iQ™ (Bio-Rad, Hemel Hempstead, UK). Detection of the fluorescence was carried out during the annealing step. Copy numbers for podoplanin and CD31 expression in the samples were determined from in-house standard curves.

### Statistical analysis

Statistical analysis was performed using the Stats Direct statistical software package and the Mann–Whitney *U* test for continuous variables. All tests were two-tailed with a 5% significance level used throughout.

## RESULTS

### Tumour growth

There was decreased tumour growth following Celecoxib treatment in both the MCF7/HER2-18 (*P*=0. 029) and MDAMB231 breast cancer cell lines (*P*=0.0003) (Table 1). These results represent the median values from a total of 43 controls and 41 treated tumours in the MCF7/HER2-18 cell line and 19 controls and 20 treated tumours in the MDAMB231 cell line. The median tumour growth inhibition (TGI) was calculated by comparing the median control growth to the individual treated tumour growth as shown by the equation: 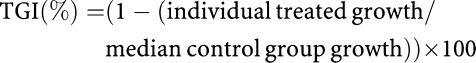


The median TGI was 58.7% in the MCF7/HER2-18 tumours and 46.3% in the MDAMB231 tumours (Table 1). No differences in weight between control and treated mice were seen.

### Celecoxib does not perturb cell proliferation

The differences in tumour growth were not a product of alterations in cell proliferation as there was no change in the median number of proliferating cells in the cell lines between the control and the treated samples as assessed by Ki67 (Table 1). Also, the p21^cip1^ pathway was not perturbed by the administration of Celecoxib, as there were no significant changes in the median number of cells in each cell line expressing p21^cip1^ (Table 1).

### Apoptosis significantly increased following Celecoxib treatment

There was a significant increase in cell apoptosis in the ER-positive MCF7/HER2-18 cell line following treatment with Celecoxib (median control TUNEL staining 0.52% (IQR 0.46–0.68), median treated TUNEL staining 0.77 (IQR 0.46–0.68) (Table 1)). There was also an increase in apoptosis in the MDAMB231 cell line that did not reach statistical significance.

### Celecoxib inactivates Akt

**Figure 1:**
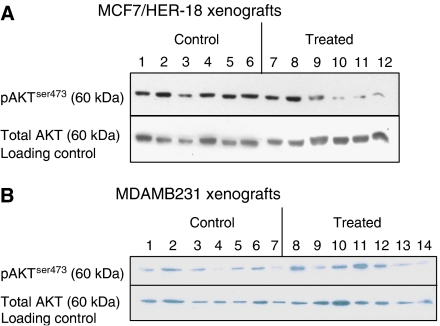
Western blot MCF7/HER2-18 pAkt^ser473^, total Akt. (**A**) Representative Western blots from MCF7/MCF7/HER2-18 control and Celecoxib-treated tumours. The top blot shows a decrease in pAKT^ser473^ in the treated samples (60 kDa). At the bottom, no change was seen in total AKT (60 kDa) as a loading control. The median arbitrary quantity from the control to treated tumours showed a decrease of 28.2% (IQR −1.5–89.1%) at densitometry over all of the tumours studied. (**B**) A representative Western blot using lysates from the MDAMB231 cell line. There was no overall pattern seen. Total AKT was used as a loading control (60 kDa), bottom blot.

**Figure 2:**
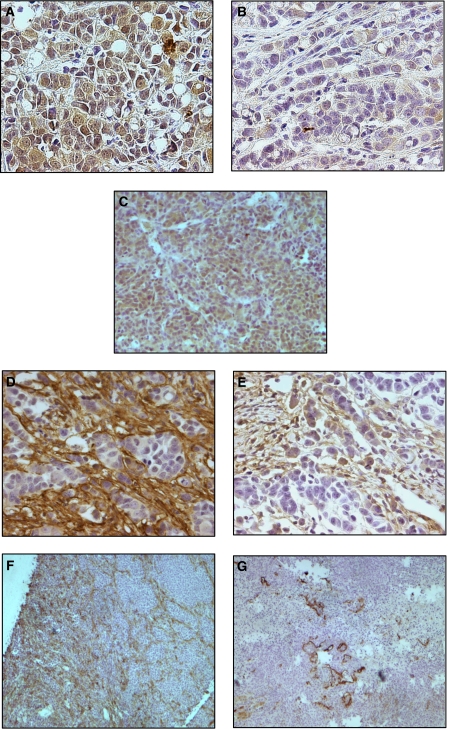
The MCF7/HER2-18 control tumours showed expression of pAKT^ser473^ in both the cell nucleus and the cell cytoplasm (**A**). Following Celecoxib treatment both the nuclear and cytoplasmic components of pAKT^ser473^ decreased, this was predominantly seen as a decrease in nuclear pPAKT^ser473^ (**B**), which decreased from a median of 57.3% (IQR 54.1–61.6) in the control tumours to 35.7% (IQR 24.4–47.6) in the treated tumours, *P*=0.0001. The MDAMB231 cell line expressed predominantly cytoplasmic pAKT^ser473^ (**C**), which did not significantly decrease following Celecoxib treatment. Decreases in lymphatic vessel density were seen in the MCF7/HER2-18 xenografts (**D** and **E**) from a median of 12 Chalkley counts to 9 Chalkley counts (*P*=0.0001). The MDAMB231 xenografts (**F** and **G**) also showed a decrease in podoplanin expression from a median of 7 Chalkley counts to 4 Chalkley counts, *P*=0.0001) following COX-2 inhibition. Representative IHC staining for pAKT^ser473^ and podoplanin in the harvested xenografts: (**A**) pAKT^ser473^ staining in MCF7/HER2-18 control xenografts (× 400); (**B**) pAKT^ser473^ staining in MCF7/HER2-18 treated xenografts (× 400); (**C**) pAKT^ser473^ staining in MDAMB231 control xenografts (× 400); (**D**) Podoplanin staining in MCF7/HER2-18 control xenografts (× 400); (**E**) Podoplanin staining in MCF7/HER2-18 treated xenografts (× 400); (**F**) Podoplanin staining in MDAMB231 control xenografts (× 100); (**G**) Podoplanin staining in MDAMB231 treated xenografts (× 100).

Akt (PKB) is a member of the serine/threonine kinases, which in its activated state, is antiapoptotic. Phosphorylation of Akt at the ser473 site was significantly decreased in the MCF7/HER2-18 cell line following treatment with Celecoxib, this was shown at both Western blotting (Figure 1A) and IHC (Figures 2A and B). In the control samples, pAkt^ser473^ was found to be in both the cytoplasmic and nuclear areas of the cell, and was distributed throughout the specimen. Celecoxib decreased both the nuclear and cytoplasmic components of pAkt^ser473^ (Figures 2A and B). The median number of nuclei that stained positive for pAKT^ser473^ dropped from 57.3% (IQR 54.1–61.1) in the MCF7/HER2-18 controls to 35.7% (IQR 24.4–47.6%); *P*=0.0001. In the ER-negative MDAMB-231 cell line, no changes in pAkt^ser473^ were seen at either Western blotting (Figure 1B) or IHC (Figure 2C), but as can be seen from the IHC sections, the distribution of pAkt^ser473^ in the MDAMB231 cell line differed from the MCF7/HER2-18 cell line in that there was very low levels of nuclear pAkt^ser473^ (Figure 2C). It therefore appears that the subcellular location of pAkt^ser473^ is an important determinant of Celecoxib signalling effect.

### COX-2 protein expression

**Figure 3:**
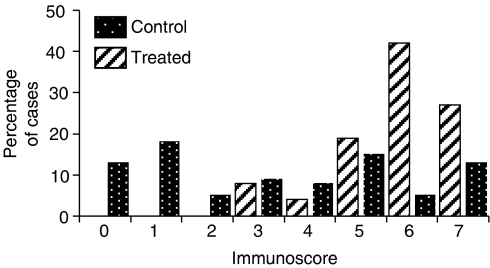
Cyclooxygenase-2 immunoscore in control and Celecoxib-treated MCF7/HER2-18 tumours. Cyclooxygenase-2 immunoscore was calculated as the sum of the intensity score 0–4 (0=none, 1=mild, 2=moderate, 3=strong, 4=very strong) and the percent positivity score (0=nil, 1⩽10%, 2=10–50%, 3⩾50%). No intensity score and therefore immunoscore could be calculated for the MDAMB231 cell line as the staining was confined in cytoplasmic vesicles.

The pattern of COX-2 expression varied between the cell lines. The MCF7/HER2-18 cell line showed homogeneous cytoplasmic staining across the majority of cells. Celecoxib treatment decreased both the median number of positive cells (median control 65.3% (IQR 60.3–77.4), median treated 22.5 (IQR 1.1–60.3)) and median staining intensity (Table 1), reflecting a significantly reduced median immunoscore (Figure 3) of 3 in the treated group, compared to 6 in the control group (*P*=0.0001). However, the MDAMB231 cell line showed staining that was confined to cytoplasmic vesicles, and therefore staining intensity could not be determined and the scoring was based solely on the number of cells that contained the vesicles. Tumours that had been exposed to Celecoxib again showed a significantly decreased staining for COX-2 protein expression (median control 26.6% (IQR 12.0–32.4), treated 9.4% (IQR 3.9–18.1) (Table 1)). In the ER-positive MCF7/HER2-18 tumours, the percentage of cells that expressed COX-2 in the cytoplasm was significantly correlated to growth; correlation co-efficient 0.4 (*P*=0.02).

### Lymphangiogenesis

Q-PCR showed a significant decrease in median podoplanin RNA from the control to the treated tumours. This was seen in both the MCF7/HER2-18 and the MDAMB231 cell lines. The median number of RNA copies per *μ*l was 66.61 (IQR 27.87–330.50) in the MCF7/HER2-18 control and fell to 2.94 copies (IQR 0.27–18.50) in the treated tumours (*P*=0.05) (Table 2). In the MDAMB231 tumours the median number of RNA copies per *μ*l was 160.65 in the control tumours (2.14–174.75) and 0.05 (IQR 0.00–2.99) in the treated group (*P*=0.015). CD31, a marker of angiogenesis also showed reduced numbers of RNA following Celecoxib treatment (Table 2). Our findings were confirmed at the protein level following immunohistochemical staining for podoplanin (Figures 2D–G). At IHC the median number of Chalkley counts in the MCF7/HER2-18 cell line fell from 12 (IQR 9—14) in the control tumours to 9 (IQR 6–10) in the treated samples (*P*=0.0001), and from 7 (IQR 5–19) in the MDAMB231 tumours, to 4 (IQR 3–6) in the treated samples (*P*=0.0001) (Table 2). At IHC it could clearly be seen that the majority of lymphatics were in clusters around the periphery of the tissue; therefore, Western blots for podoplanin were not performed.

## DISCUSSION

### COX-2 inhibition decreases tumour growth

High rates of tumour growth and metastasis lead to death from breast cancer. In the nude mice, median tumour growth was inhibited by 58.7% in the ER-positive MCF7/HER2-18 tumours and by 46.3% in the ER-negative MDAMB231 tumours, following treatment with Celecoxib. Oestrogen receptor-negative tumours are unresponsive to hormonal manipulation, by way of aromatase inhibitors and tamoxifen, and conventional treatment options are therefore limited. Perturbing the COX-2 signalling pathway may prove to be an important novel therapeutic strategy in both ER-positive and ER-negative tumours reducing the rate of tumour growth.

### Celecoxib increases apoptosis via inactivation of Akt

The mechanism of action of Celecoxib in inhibiting tumour growth was by increasing apoptosis. In the MCF7/HER2-18 cell line, the median number of cells showing apoptosis increased by 40% from the control to the treated tumours. The fine balance between apoptosis and proliferation is crucial for regulating growth; therefore, even the small numerical changes in apoptosis translate to a large percentage difference, and a significant effect on growth. There were no discernable changes in cell proliferation. This has important implications for current and future trials utilising Celecoxib in breast cancer, as apoptosis, not proliferation should be used as the primary endpoint. Mechanistically, the increase in apoptosis was mediated by inactivation of Akt in the MCF7/HER2-18 cell line. The decrease in pAkt^ser473^ that we demonstrated was predominantly nuclear with a median decease of nuclear expression from 57 to 36%. Akt (also known as PKB) is a serine threonine kinase that acts downstream of PI3 kinase in the prosurvival pathway. It is thought that activation follows recruitment to the plasma membrane and phosphorylation of both its serine and threonine residues. It has been demonstrated that within 30 min of activation, Akt detaches from the cytoplasm and relocates to the nucleus. In the nucleus there is modulation of the phosphorylation of a variety of transcription factors (Andjelkovic *et al*, 1997), including phosphorylation of FKHR1 (Rena *et al*, 1999) and the forkhead transcription factor AFX (Kops *et al*, 1999) promoting their cytoplasmic retention – distancing them from their nuclear targets. It has previously been shown that the growth factors PDGF and IGF-1 both cause an increase in the amounts of intra nuclear Akt (Borgatti *et al*, 2000). There is still, however, some debate as to whether activation occurs solely in the cytoplasm with subsequent translocation to the nucleus, or whether there is a nuclear component to the event itself (Borgatti *et al*, 2000). The subcellular localisation of pAkt is therefore important for its signalling effect which in the MCF7/HER2-18 cell line was both cytoplamic and nuclear, but predominantly cytoplasmic in the MDAMB231's. This lack of nuclear activated Akt could explain why there was no decrease in phosphorylation seen in the MDAMB231 tumours (and a less substantial increase in apoptosis). Reduced Akt activation following Celecoxib treatment has also been seen in prostate (Hsu *et al*, 2000) and hepatocellular (Leng *et al*, 2003) cancer cells. The importance of Akt inactivation in increasing apoptosis was confirmed in PC-3 prostate cancer cells that were engineered to constitutively overexpress Akt which showed reduced apoptosis following Celecoxib treatment compared to cells without constitutively active Akt (Hsu *et al*, 2000).

### Celecoxib reduces lymphatic vessel formation

Celecoxib reduced levels of the lymphatic endothelial cell marker, podoplanin RNA by 87% in the MCF7/HER2-18 and 99.9% in the MDAMB231 tumours. The control samples showed variable levels of podoplanin RNA (as seen by the interquartile range values), showing that lymphatic response is specific to the individual tumour/host. Following Celecoxib treatment this range was much less variable, with in some cases a complete lack of expression of podoplanin RNA. The decrease in expression was also seen at a protein level following Chalkley vessel counts; where the lymphatic vessels were predominantly clustered around the tumour periphery. In addition, there was a reduction in the median number of RNA copies of CD31 (an angiogenic marker) by 84% in the MCF7/HER2-18 and 91% in the MDAMB231 tumours. The antiangiogenic properties of the COX-2 inhibitors have already been demonstrated (Masferrer *et al*, 2000). As CD31 and podoplanin RNA were both greatly reduced in the MDAMB231 tumours, this may account for the fact that there was decrease in growth of the MDAMB231 tumours following Celecoxib, even though the increase in apoptosis failed to reach statistical significance. Review of the IHC sample sections showed large areas of central necrosis in the MDAMB231-treated tumours, which again would be consistent with an insufficient blood supply in these Celecoxib-treated tumours.

In breast cancer the primary mode of metastasis is via the lymphatics and COX-2 expression correlates with lymph node metastasis in breast cancer (Ranger *et al*, 2004). Taken altogether the cumulating data regarding COX-2 inhibition and lymphangiogenesis indicate that there is interesting potential of COX-2 inhibitors being used as antimetastatic agents.

### Celecoxib decreases COX-2 protein expression

Celecoxib treatment decreased the levels of COX-2 protein expression in both cell lines. Therefore, Celecoxib is not simply blocking the effect of COX-2, but is also perturbing COX-2 protein production; a finding noted in previous studies (Cheng *et al*, 2002; Agarwal *et al*, 2003). In COX-2 expressing HT29 colon cancer cell lines, COX-2 inhibition with another selective COX-2 inhibitor SC236 (a structural analogue of Celecoxib) showed that at high levels of inhibitor (concentrations greater than 75 *μ*m), COX-2 enzyme activity was completely inactivated. Decreased levels of COX-2 protein expression and increases in apoptosis were seen. Whereas Sulindac sulphide (an inhibitor of COX-1 and COX-2) did not promote any changes in apoptosis and also showed no decrease in COX-2 protein expression) (Agarwal *et al*, 2003). The antiapoptotic effects of COX-2 were better correlated to decreases in COX-2 protein expression than inhibition of enzymatic activity (Agarwal *et al*, 2003). It has been suggested that the prostaglandin products of COX-2, mainly PgE2, positively feed back to produce enhanced COX-2 protein expression (Faour *et al*, 2001). Therefore blocking COX-2, and hence prostaglandin production, would decrease this positive feedback loop. NF*κ*B also regulates the COX-2 gene. Celecoxib abrogates TNF-induced activation of NF*κ*B with inhibition of Akt (Shishodia *et al*, 2004), and therefore another possible explanation for the decrease in COX-2 protein levels seen could be that there is inhibition of NF*κ*B-induced promoter activity of the COX-2 gene, with subsequent decreases in COX-2 protein expression.

### COX-2 independent mechanisms of Celecoxib action

In the ER-positive MCF7/HER2-18 treated tumours, the percentage of residual COX-2 expression significantly correlated with tumour growth, but in the ER-negative MDAMB231-treated tumours, there was no correlation between residual COX-2 expression and growth. This raises the possibility of COX-2 independent effects of Celecoxib in these ER-negative tumours. Further work needs to be carried out to explore the possible COX-2 independent effects of these inhibitors and possible different modes of action in ER-positive *vs* ER-negative cases. New generation products, which are structurally similar to the COX-2 inhibitors, but lack COX-2 inhibiting effects, are currently under development.

### Future direction

We have shown that Celecoxib significantly decreases breast tumour growth in this nude mouse model and clinical studies are underway to investigate the effect in patient populations. Cyclooxygenase-2 inhibitors are showing great promise for their potential therapeutic benefit in a range of cancers; however there has been recent concern over potential cardiotoxicity. This was due to an increased risk of cardiovascular events following treatment with another COX-2 inhibitor Rofecoxib (Vioxx® – now withdrawn) that was highlighted following the publication of the APPROVe trial (Bombardier *et al*, 2000; Bresalier *et al*, 2005). In 2005 the *New England Journal of Medicine* published a review of Celecoxib cardiac safety which raised concerns over prolonged high-dose COX-2 inhibition (Solomon *et al*, 2005). A review of the data by Kimmel *et al* (2005) stated that patients who had been taking Rofecoxib had a three times greater risk of developing a myocardial infarction than patients taking Celecoxib, but the overall safety of Celecoxib is still under debate. As Celecoxib has now been shown to significantly decrease tumour growth, there is an urgency to develop new inhibitors that do not have the potential cardiotoxicity. We must maintain the momentum for further investigation of this class of drug, and its descendents, in relationship to their chemotherapeutic potential and mechanisms of action.

In summary, Celecoxib significantly decreased ER-positive and ER-negative breast tumour growth in our nude mouse xenograft model. The mechanism of action was via inactivation of AKT permitting increased cell death in the ER-positive tumours. The primary mode of breast tumour spread is via the lymphatics. Increasing evidence suggests that COX-2 has an important role in lymphangiogenesis and we have shown that COX-2 inhibition decreases new lymphatic vessel formation with the potential decrease in metastatic spread of both ER-positive and ER-negative breast tumours.

## Figures and Tables

**Table 1:** Tumour growth, proliferation and apoptosis in the cell lines studied

	**Cell line**					
	**MCF7 HER2/18**			**MDAMB231**		
	**Control**	**Treated**	***P*-value**	**Control**	**Treated**	***P*-value**
Median growth (mm^3^)	100.8 (38.4–183.5)	39.3 (4.0–156.1)	0.029	96.4 (51.3–284.4)	29.7 (0.0–53.3)	0.0003
Median tumour growth inhibition (%)	58.7 (−7.3–98.2)			46.3 (3.7–97.4)		
Mouse weight gain (g)	0.1 (0.7–1.2)	0.8 (0.1–1.6)	0.08	1.9 (1.7–2.0)	2.2 (1.7–2.6)	0.22
Median Ki67 (%)	55.0 (50.9–68.9)	55.5 (47.8–63.0)	0.65	55.1 (47.2–71.6)	57.9 (47.7–60.0)	0.91
Median p21^cip1^ (%)	14.5 (12.1–17.0)	16.8 (13.1–20.9)	0.07	0.6 (0.2–2.9)	0.3 (0.1–0.3)	0.88
Median TUNEL (%)	0.52 (0.40–0.63)	0.73 (0.58–0.85)	0.0004	0.51 (0.47–0.82)	0.56 (0.343–0.98)	0.96
Median percentage COX-2 positive cells	65.3 (60.3–77.4)	22.5 (1.1–60.3)	0.0001	26.6 (12.0–32.4)	9.4 (3.9–18.1)	0.0001
Median COX-2 staining intensity^a^	3+	1+	0.046	—	—	—

a No staining intensity values given for the MDAMB231 cell line as the COX-2 protein was confined to vesicles which could not be quantified by intensity.

**Table 2:** Q-PCR for lymphangiogenic (podoplanin) and angiogenic (CD31) markers and Podoplanin immunohistochemistry (IHC) Chalkley vessel counts

	**Cell line**					
**Median RNA copy per *μ*l (IQR)**	**MCF7/HER2-18 control**	**MCF7/HER2-18 treated**	***P*-value**	**MDAMB231 control**	**MDAMB231 treated**	***P*-value**
Podoplanin	66.61 (27.87–330.50)	2.94 (0.27–18.50)	0.05	160.65 (2.14–174.75)	0.05 (0.00–2.99)	0.02
CD31	47.48 (24.34–56.08)	7.70 (4.50–21.18)	0.08	140.30 (33.84–581.71]	12.87 (3.57–59.69)	0.09
Median Podoplanin IHC Chalkley vessel counts	12 (9–14)	9 (6–10)	0.0001	7 (5–19)	4 (3–6)	0.0001
